# Self-Assessment of Students of Geography Education and Primary Social and Science Teaching towards the Use of Digital (Geo-) Media for Written and Oral Argumentation

**DOI:** 10.3390/ejihpe12060038

**Published:** 2022-05-25

**Authors:** Sebastian Wolff-Seidel, Alexandra Budke

**Affiliations:** Institute for Geography Education, Faculty of Mathematics and Natural Sciences, University of Cologne, Gronewaldstraße 2, 50931 Cologne, Germany; alexandra.budke@uni-koeln.de

**Keywords:** argumentation, higher education, digital geomedia, open educational resources, OER

## Abstract

This article discusses possible challenges and potentials in the use of digital geomedia in the context of written and oral argumentation in higher education by examining the perspectives of students and lecturers, especially for the subjects of geography in general and primary school science. To this end, potentials and challenges, as well as competences that have to be promoted among pupils and students in dealing with digital geomedia in the context of written and oral argumentation are first discussed. In the following, we present the results of a mixed methods approach in which we surveyed student teachers with a questionnaire (*n* = 150) on the one hand and, on the other hand, reflected their view of the issue by analysing qualitative expert interviews (*n* = 17) with lecturers who teach at the same universities in the corresponding degree programmes. In this way we contextualise the student’s self-assessment in the respective location’s teacher training. Our results show that there is a high degree of commonality between lecturers and students with regard to the importance of argumentation with digital geomedia. At the same time, the assessment of the students’ abilities differs greatly; most students feel capable of dealing with these topics, while lecturers see deficits here.

## 1. Introduction

Pandemics, migration, resource scarcity, and international conflicts are all issues characterised by their complexity, controversial nature, and a multitude of different positions. New media, particularly the internet, are full of information about these topics, the majority of which is current, and can provide a wide range of spatial data and offer the possibility of comparison between locations. In addition, these issues share the characteristics of having a relevant spatial component. In this regard, geography lessons have the potential to make an important contribution to the political education of pupils and the opinions they form by showing them how to obtain information and how to take a meaningful position in social debates. As a result, argumentation skills also play a central role in enabling pupils to understand arguments in these conflicts and to formulate their own positions and participate in discourse. However, the internet also poses great challenges for learners due to the uprising of “fake news” and the huge amount of information available.

When considering the above, the question arises as to how to deal with these challenges. Studies show that pupils have difficulty finding geographical information on the internet [1] and studies on subject-specific implementation show that teachers struggle to develop pupils’ political argumentation skills and creative competence in geography lessons [2]. In addition, findings on the production and reception of arguments show that pupils and geography students have major problems with the production and reception of geographical arguments [3]. So far, there is no knowledge regarding the assessment of future teachers (students) in relation to the possibilities of promoting argumentation skills with the help of digital (geo)media in geography lessons. This work subsequently addresses this gap and relates the students’ self-assessment to the assessment of the lecturers in their degree programmes. The results of this study can provide an approach to whether and how support materials can and should be used to support students in these areas.

The research results presented here were generated on the fringes of the Federal Ministry of Education (BMBF) joint project DiGeo. This project serves to develop and research the generalizability and transferability of digital subject concepts using the example of digital geomedia use in teacher education to promote the 3 areas: Argumentation & Communication, Reflection & Reflexivity, and Participation & Design.

In the following sections we present the current state of research on argumentation and new media in relation to geography education, then look at the potentials of and challenges in using digital media in the context of argumentation. Afterwards, we present the results of a mixed-method approach in which we firstly used a standardised online survey among students (*n* = 150) of geography and primary social and science education at four German universities, to subsequently triangulate these with the results of qualitative expert interviews with a total of 17 lecturers from the corresponding study programmes of the respondents. The comparison of the lecturers’ statements and the learners’ assessments provides new insights into the attitudes and self-assessments of students and lecturers of geography towards the use of geomedia in the context of argumentation in higher education. This led to three research questions:

- What importance do students and lecturers attribute to argumentation with geomedia in their studies respective to their courses?
- How do students assess their own abilities to argue with geomedia and how are these assessed by lecturers?
- How do lecturers promote students’ competences in argumentation with geomedia in the current study of geography or subject teaching?

## 2. State of Research/Theoretical Background

Currently, there are numerous publications on both argumentation competencies and digital geomedia that deal with use and training in these areas. At this point, we present approaches to promotion in both areas. We then describe the potentials and challenges that exist in the attempt to promote argumentation skills with the help of digital geomedia.

### 2.1. Argumentation and Argumentation Skills

Many topics in geography lessons are complex and are characterised by controversial viewpoints and debates on the use of space, which is why the topic of argumentation is relevant to geography lessons. In contrast to explanations, in the case of argumentation the facts, theses, and connection between both are controversial [4]. Conflicts over the use of space are characterised by a high degree of complexity, which is formed by various interrelated positions or elements that operate at different spatial scales and move within a spatio-temporal dynamic [5] (p. 177). As a result, in geography it is often not possible to find a single correct solution or argument as the assessment of individual arguments may differ from the point of view of the arguing party. Instead, different positions must be weighed against each other, and solutions characterised by compromises. From a systematic point of view, a distinction can be made between oral and written argumentation skills, and subsequently between the dimensions of reception, production, and interaction of both [6] (p. 184).

Argumentation can enrich the subjects of geography, primary science, and social education in many ways [7]: First and foremost, it can promote a general understanding of how science works [8,9]. It also contributes to the political education and maturity of learners by developing their understanding of foreign positions (reception) and to promote development of their own positions (production) and to represent them (interaction), which is important as several studies have shown that many students and schoolteachers have poor argumentation skills [10,11,12]. Further studies have shown that in class pupils tend to emphasize their own position and argue for it in discussions and controversial topics and not considering or appreciating opposing positions adequately [13] (p. 76). This is contrary to a multi-perspective approach, which is necessary in geography lessons. This also applies to geography student teachers, for whom deficits were also identified in this area [14,15]. These deficits are particularly noteworthy because the study by Lytzerinou and Iordanou [10] showed that the competence of teachers to write argumentations correlates with the ability to assess pupils’ performance in this area. These deficits around written argumentation appear to be widespread, as analyses of written argumentation by student teachers have shown that they generally have little coherence in their own argumentation skills [16]. While there do exist approaches to counteract these deficits [17,18], these do not yet refer to concrete methods using digital media. To deal with the challenges in the use of digital geomedia in the context of argumentation, the following section will look at digital geomedia.

### 2.2. Digital (Geo-)Media

“*Geomedia—in short, every media that involves geo-referenced information—is an essential part of everyday life.*” [19] (p. 182). Digital (geo)media can play a central role in learning about geographical conflicts, as they can be used to retrieve information about the conflict, examine the positions of actors, and interactively exchange arguments. This applies not only to maps, atlases, and globes, but also to all other media such as images, texts, films, drawings, or animations, which store and transmit geospatial information [20] (p. 210). Map services can show borders, satellite images can visualise the ways in which space is used and can be used to compare different sources, and texts can reveal quite different points of view on spatial topics. The use of digital geomedia is characterised by its topicality, the availability of a variety of different sources and the possibility of interaction between locations [21]. This means that pupils can look up updated information on conflicts over the use of space daily, which is particularly useful as these types of conflicts have a high level of spatio-temporal dynamics.

The possibility to access various sources for example in the form of the homepages or social media sites of governments, NGOs, companies, scientific institutions, or news providers enables the user to explore conflicts from multiple perspectives, and to get to know different ways of argumentation. Furthermore, there is the possibility to exchange ideas, and to contact different actors (e.g., citizens’ initiatives or NGOs) and get to know their point of view directly. Web 2.0 also offers pupils the possibility of playing themselves in digital media and argumentation by producing their own maps for exchanging views, and consequently for active argumentation. In comparison, the limitation of textbooks is that, whilst they provide credible and relevant information through the selection of authors, they often do not contain up-to-date information and quickly become outdated as a result, and consequently contain errors or lack information for use in argumentative and multi-perspective tasks [22] (p. 261).

Spatial developments and conflicts, which are often the subject of geography lessons, can therefore be better understood using digital geomedia [23] (p. 233); [24] (p. 73). In addition, students are generally more enthusiastic about the use of digital media in their studies [25,26]. However, the use of digital media also poses a challenge. Young people have considerable problems when searching and evaluating information online [27] (p. 77).

It has been found in previous research that whilst German teachers often use digital media to a limited extent, they are open-minded towards its use [28]. Teachers of geography often find it difficult to reflect on spatial constructions and their geomedia representations [29], such as ways of representing and describing spaces with which power relations are consolidated and legitimised. They consequently pass these difficulties on to their students.

For pupils, it has been found that they often do not take a critical view on information gathered from the internet [30] (p. 236). Pupils also have difficulties in correctly differentiating between news media and facts and advertising [31] (p. 10). Studies on pupils’ behaviour when undertaking internet research on complex geographical issues show that pupils make limited use of map services and pay little or no attention to the topicality of information and websites [1]. Despite these difficulties in dealing with digital media, further studies have shown that pupils rate their own skills in dealing with digital media as high [32] (p. 205); [33] (p. 15).

### 2.3. Challenges and Potentials in the Use of Digital Geomedia in the Context of Argumentation

Argumentation requires the conclusive proof of theses/assertions with the help of facts [4]. Digital geomedia can therefore play a useful role regarding the use of argumentation in geography lessons, not only when drafting arguments but also by enabling multi-perspectivity, showing developments through comparisons and providing constantly updated information [34]. Digital (geo)media provides a variety of information on a daily basis, which, when used reflectively and reflexively, can help us to develop our own positions in public debates and can therefore be important for the development of our own argumentative standpoints. This is where, for example, the “education for spatial citizenship” approach comes in, which aims to promote a reflexive use of digital geomedia both in the use and in the construction of geomedia. This approach aims to educate citizens in the use of digital geomedia so that they can successfully participate in a democratic society and is based on a foundation of technical-methodological principles, ability to communicate, participate and negotiate with the help of geomedia, and cognitive ability to reflect and be reflexive in dealing with geomedia. Combining technical-methodical skills when dealing with geomedia in political education, where the fields of communication, participation, and negotiation, among other things, play a role, forms a link to argumentation, which in turn is a component of communication, and particularly negotiation, as the exchange of arguments is central to negotiation [35,36].

**Figure 1:**
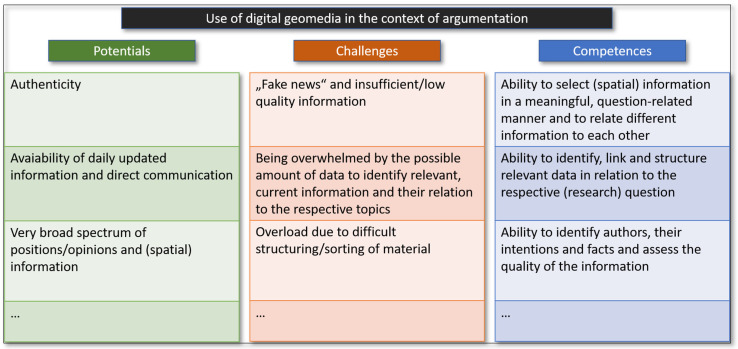
Potentials, challenges & competences for the use of digital geomedia in the context of argumentation, own figure.

As explained previously, the use of digital (geo)media in the context of argumentation offers potential and challenges, and consequently requires the learning of skills or competences. We will briefly outline these in the following model (see Figure 1). The fact that the internet is extremely widespread and gives almost everyone the opportunity to express their opinion also means that, in principle, a much more comprehensive picture of the spectrum of opinions can be displayed or researched. This contrasts with textbooks, whose authors may restrict the discourse to fewer perspectives. The so-called “double-page principle” is particularly applicable to textbooks in Germany, where each double page in a textbook presents an entire topic and, due to the available space, often have a limited and not multi-perspective view on these topics as a result. In addition, a larger number of different sources and information can be used, and consequently comparisons can be made between them. Finally, there is the possibility of obtaining authentic information directly from the sources, which is not necessarily the case with textbooks in Germany, for example, where fictional characters are often used to depict conflict positions [37]. This leads us to the consideration of multiperspectivity, which is a central quality criterion for argumentations in the subject of geography. Arguments are of high quality when spatial perspectives and multiperspectivity are considered and are justified [38] (p. 276). As a result, digital (geo)media can offer more authenticity and thus establish a concrete connection between reality and everyday life, as well as an insight into multi-perspectivity, because different points of view can be accessed. However, authenticity does not necessarily mean information is factually correct (keyword “fake news”) so pupils must learn to identify authors and facts. Another consideration is the constant updating and consequent topicality of the content, especially in contrast to textbooks. As topicality is a central teaching principle in geography lessons [39] (p. 172), digital media as source material has a clear advantage. Moreover, German geography textbooks are not updated frequently enough to keep up with current developments and show shortcomings in terms of educational standards because they rarely contain argumentation tasks [22] (261). In contrast, the challenge of using digital offerings lies, among other things, in the identification of appearance data and the link to the dynamically occurring process of the geographical problem or the conflict of spatial use. Nonetheless, digital media offers a point of connection between informal learning processes and those in teaching and studying, and thus a concrete, authentic reference to life and current events that goes beyond the possibilities of textbooks [40] (p. 22). In addition, the use of digital media enables in-depth, cross-location or location-independent interaction, so that it is possible to work with information from different locations and actors in conflicts focused on the use of space can be questioned directly about their interests and motivations. The technology can therefore be very helpful in analysing arguments by interviewing authentic sources in real time and analysing their positions. However, ultimately, it should also be noted that when digital (geo)media is used, the goals and contents pursued remain the same because the same methodological model concepts are also followed when dealing with argumentation, and consequently success depends primarily on the conception of the learning offer [41] (pp. 77–111). A challenge in this area arises from a possible overload of information due to linguistically heterogeneous texts and language barriers.

The wide range of information available digitally holds both potential and challenges. The challenge arises from the large quantity of information that has to be filtered, targeted to the question-related information, and compiled in a meaningful, structured way [1].

Finally, and specific to geography, this multitude of digital information also offers a comprehensive range of concrete spatial information at a wide variety of scales. Again, the challenge is to identify concrete question-related information, and students must learn to identify such information correctly with the correct scale and spatial reference. In summary, the use of digital geomedia offers a wide range of possibilities and real advantages for dealing with the teaching of argumentative skills in geography lessons, but it also poses challenges. In the following section we take a closer look at the attitude and self-assessment of future teachers towards digital (geo)media, argumentation, and the use of digital geomedia in the context of argumentation as well as on the perspective and assessment of lecturers of these same students.

## 3. Materials and Methods

As formulated in the introduction, the aim of this article was to discuss possible challenges and potentials in the use of digital geomedia in the context of written and oral argumentation in higher education, by looking at the perspectives of students and teachers, especially for the subjects of geography in general and science in primary school.

Methodologically, we proceeded in three steps: first, to survey the importance that students and teachers in geography attach to the use of argumentation with digital geomedia; second, to investigate how students themselves assess their competences in relation to the use of argumentation with digital geomedia and how teachers assess these competences; and third, with regard to teachers, to survey how they promote their students’ competences in these areas. For this purpose, a mixed-methods approach was developed in which a quantitative and a qualitative sub-study were conducted and directly related to each other in the form of a sequential multi-method design [42] (p. 27).

**Figure 2:**
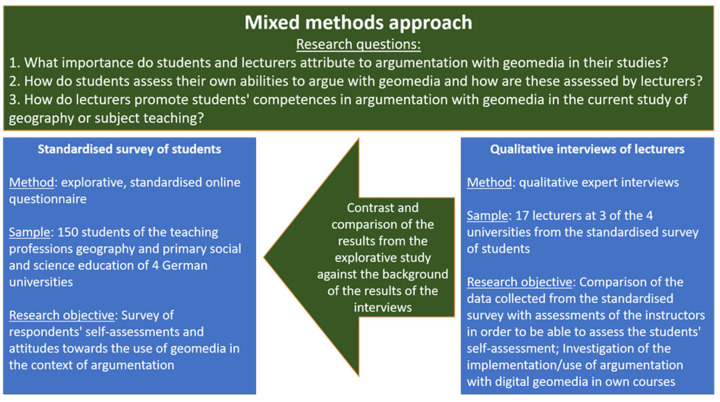
Illustration of the methodological procedure in the course of the method mix, own figure.

The first step in this approach was to collect the perspective of the students themselves in an explorative questionnaire study. Students in teacher training courses of Geography and primary social and science education at a total of four German universities (Duisburg-Essen, Frankfurt am Main, Cologne and Wuppertal) were surveyed using a quantitative online survey. In addition, 17 qualitative interviews were conducted with lecturers at three of these universities. These interviews were conducted together with colleagues from the BMBF joint project DiGeo at the respective universities. Due to the cooperation in the DiGeo joint project, the interview guideline used contained further question blocks on the partners’ topics, but for this study only the question blocks on digital media and argumentation as well as the query on teaching experience and activity are taken into account. This was helpful in acquiring the interviewees. The interview guide was the same for all interviews to ensure comparability. Due to the Covid19 pandemic, all interviews were conducted digitally via video conference. With the help of the results of these interviews, the results of the quantitative survey were then contrasted and compared (Figure 2):

In the following, we introduce the samples of the two parts of this study, then present the survey instruments used and take a critical look at the approach.

### 3.1. Participants and Samples

For the explorative questionnaire study, students at a total of four German universities (Duisburg-Essen, Frankfurt am Main, Cologne and Wuppertal) were surveyed. The population of this survey consisted of the students enrolled in the degree programmes for teaching geography and physical education at the four universities studied during the survey period in the summer semester of 2020. A total of 150 fully completed questionnaires could be evaluated. Unfortunately, a total number of 33 questionnaires had to be sorted out that had only been processed very incompletely. With regard to the high drop-out rate, we assume that many surveys were taking place at the same time, which is why many students did not have the motivation to complete the questionnaire. The proportion of students at the location with the highest response rate (Cologne) was 17 percent in relation to the number of students enrolled there. At the other locations, the participation rates were in part significantly lower (Duisburg-Essen 6.67%, Frankfurt am Main 3.64%, Wuppertal 2.5%). In this respect, the results presented here can hardly be considered representative for all students of those subjects in Germany, but in combination with the qualitative interview study they may provide the opportunity to derive explorative theses and at least allow statements to be made about the situation at four German universities in the study programmes geography and primary social and science education. With a proportion of 73%, significantly more of the respondents were female than male with 27% proportion. The average age of the respondents was 24 years, with a median of 23 years and a standard deviation of 5.67. 68% of the respondents were between 20 and 25 years old. On average, the respondents were enrolled in the 5th semester, with a median value of 4 and a standard deviation of 3.79% of the respondents had been enrolled for a maximum of 6 semesters. 62% of the respondents were pursuing the bachelor’s degree, 21% the master’s and 17% were enrolled in the Staatsexamens program (Frankfurt). In terms of the study completion target, around 59% of those surveyed were students aiming to become teachers at Hauptschule, Realschule, and Gesamtschule (Hauptschulen and Realschulen are types of secondary schools in Germany, Gesamtschulen are comprehensive schools, also a type of secondary school, Gymnasien are also secondary schools with the explicit aim of acquiring the higher education entrance qualification. However, this is also possible at comprehensive schools. In North Rhine-Westphalia, Hauptschulen and Realschulen are attended from grade 5 to grade 10, Gymnasien up to grade 13. This is also possible at comprehensive schools, where, however, qualifications can also be obtained after the 10th grade), 28% were students aiming to become primary school teachers, 12% were studying to become Gymnasium teachers and 1% were studying to become teachers at special schools.

To complement and ultimately contrast and compare the results, guided interviews were conducted with lecturers at the universities of Duisburg-Essen, Frankfurt am Main, and Cologne. A total of 17 interviews were conducted. The decisive factor for the selection of the experts was their actual teaching activity or experience in geography, respectively, primary social and science education (the interviewees had an average of 10 years of teaching experience at universities, ranging from 3 to 26 years). In addition, care was taken to select lecturers from different sub-fields of geography (physical geography, human geography, cartography, and didactics of geography) in order to obtain as broad an overview as possible of the sub-areas of the discipline and, if necessary, to ascertain different perspectives and approaches.

### 3.2. Methodological Approach: Triangulation of Quantitative Questionnaire and Expert Interviews

We designed a survey in the form of a quantitative questionnaire using Limesurvey. In preparation for a larger-scale survey, a pre-test was first conducted with a sample of student teachers (7 respondents) to optimise the comprehensibility and answerability of the questions. The online survey was then distributed via the Institutes for Geography, Didactics of Geography and Subject Teaching at four German universities (Duisburg-Essen, Frankfurt am Main, Cologne and Wuppertal) using institute distribution lists and newsletters, and by explicitly inviting student teachers to participate. The target group of this study was therefore geography and primary social and science education (the subject within which geography is taught in primary schools in Germany) student teachers. It should be noted that in the cases of the universities of Duisburg-Essen and Wuppertal only student teachers studying primary science and social education were included in the survey, whereas at the universities of Cologne and Frankfurt am Main students from all school forms were surveyed. The survey period was April–May 2020. The survey included the collection of personal data to determine independent variables that might influence respondents’ attitudes (age, gender, place of study, course of study, subjects, number of semesters). Three blocks of questions were then used, first to assess learners’ attitudes towards digital media, then in relation to argumentation, with a final block of questions to assess respondents’ views and self-assessment on the importance of digital geomedia for use in the context of argumentation. We used Likert scales to estimate the attitude of the students (cf. Table 1):

Since this was primarily an exploratory study with the aim of collecting the self-assessment of the students, only descriptive analyses of the data were carried out in order to be able to contrast and compare them with the interview statements of the lecturers in the sense of the method mix. For the interviews with the 17 lecturers, an interview guideline was developed [43] (p 193) which was intended to classify the results of the student survey and to compare them with the view of the lecturers, who on the whole have another assessment of the abilities of their students than the students themselves. An interview guide was developed and appointments were made with the interviewees (cf. Table 2). All interviews were conducted in German, the quotations reproduced here are translations by the authors. The average interview length was 49 min with an average variation of 14 min per interview. The interviewees were assured of anonymity in order to reduce inhibitions and to create as open an atmosphere as possible. The aim was to create the most collegial discussion situation possible in order to collect the individual views of the interviewees as uninhibitedly as possible. The following table gives an overview of the interview guide (Table 2):

The data was then analysed, whereby the interviews were first anonymised and transcribed, with a slight linguistic smoothing. The text material was analysed using software (QCAmap) and the procedure of structuring qualitative content analysis, whereby the categories were formed deductively on the basis of the theoretical considerations (cf. Figure 1). In order to further reduce bias in relation to the analysis of the interviews, they were analysed independently by the authors of this study until the highest possible intercoder reliability could be achieved. Accordingly, the following five main categories were formulated for analysis: 1. potentials in the use of digital geomedia in the context of argumentation; 2. challenges in the use of digital geomedia in the context of argumentation; 3. skills and competences that students need to learn in the use of digital geomedia in the context of argumentation; 4. skills and competences that students already have and 5. the use and implementation in the training of future teachers in higher education. The following table gives an overview of the analysis categories used (Table 3):

### 3.3. Research Limitations

Regarding the questionnaire, the low response rate of ca. 7% cannot be regarded as representative of the total number of students in the teaching offices for geography and primary social and science education in Germany. It cannot be assumed, even with the use of the institute newsletters, that all students were reached. Another explanation for the low response rate could be, among other things, that during the survey phase in the wake of the coronavirus pandemic, a lot was moved to the digital domain overall and an extremely high number of studies were conducted online. In this respect, an overall overload among the respondents can be assumed. In addition, filling out the questionnaire may have been too time-consuming overall. Nevertheless, the exploratory research design allows derivation of hypotheses from the results obtained and at least makes it possible to make statements about the sample studied. Furthermore, the mixed-methods approach aimed to contrast the students’ statements against the background of the lecturers’ assessments in precisely these study programmes. Although it is not possible to describe one perception as the “right” one, by contrasting the assessments of students on the one hand and lecturers on the other, a clearer picture can be gained.

For this survey, it should be noted that the surveys took place in early 2020, when the developments and consequences of the COVID pandemic were not yet be known. The development of digital learning formats has subsequently accelerated at German higher education institutions during the pandemic, and digital communication and learning formats have gained significantly in importance. In this respect, it can be assumed that if students were now surveyed, they may be more open-minded, particularly due to the greater number of experiences in dealing with such digital formats.

## 4. Results

The following section presents the results of the triangulated method mix consisting of the interview statements of the lecturers and the results of the student survey. We always start by showing students’ self-assessments and then contrast these with the results of the qualitative interviews using anchor quotes. Here we focus on three main areas, which serve to answer the three questions guiding the research: (1) assessment of the importance of argumentation with geomedia by the lecturers and students, (2) assessment of the students’ own skills in relation to argumentation with geomedia and assessment of these skills by the lecturers, and (3) concrete implementation and promotion of such skills by the lecturers in their courses.

### 4.1. Assessing the Importance of Argumentation with Geomedia

With regard to the importance of argumentation with geomedia, statements in the category “potentials” that were particularly meaningful and show that many of the lecturers consider the importance of argumentation with geomedia to be very significant. This lecturer reports on a session in the context of a cartography and GIS course that students here are to learn an active handling of geomedia and also learn to create them themselves, whereby the aim is to use the cartographic means specifically in such a way that one’s own position is supported in an argumentation:

“My introduction to geographical information systems always alludes to the fact that if you can create maps yourself, visualise data and use it, you can then make your own point of view much clearer, i.e., you can fulfil this level of argumentation much better.” (I_Col_No5)

Most lecturers, however, expressed less integrative approaches and referred only to the benefits of using geomedia or to argumentation. Accordingly, the aspect of authentic, up-to-date, and particularly impressive possibilities for visualisation with the help of digital geomedia was also emphasised by lecturers at other locations, as the following two quotations show:

“The opportunities are clearly the visualisation of geographical facts, for example. So, if I can use Google Earth, for example, or “GoogleStreetview” to look directly into a favela in São Paulo, for example, and see the situation on the ground, or if I can use Google Maps to look at satellite images of the tropical rainforest, for example, and see the changes in rainforest deforestation over time, then of course these are great tools for illustrating something.” (Int_FfM_No1)

Nevertheless, there were also statements that emphasised, for instance, the need to work with geomedia in the course of studies in order to strengthen the students’ argumentation reception skills and thus provide them with an understanding of social discourses: “And with geomedia, yes, of course you also have to address examples of geomedia, if they are texts from social media or pictures, then you can work on them great. I think it’s a great way to look at discussions and how you can or should or shouldn’t deal with them. I think it’s a great opportunity.” (Int_FfM_No3)

Thus, while in digital geomedia especially the visual advantages were named, and in argumentation production and reception competences were addressed, but not interaction, it can be stated overall that only few integrating statements were made as a whole, but the two parts were largely thought of separately.

**Figure 3:**
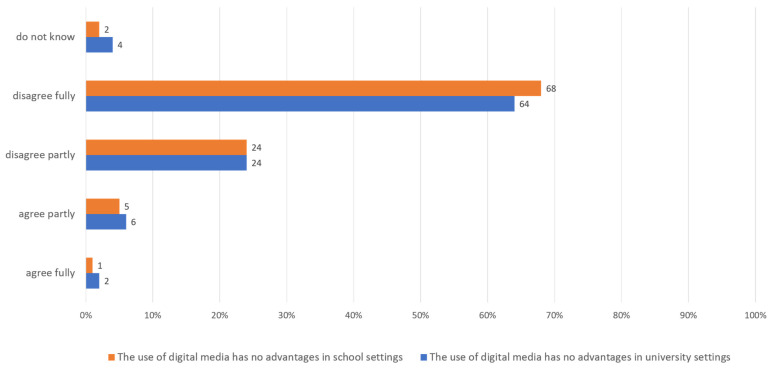
Students’ (*n* = 150) agreement with selected statements regarding the importance of argumentation in teaching and studying, own figure.

The overall very positive attitudes of the lecturers towards the field of argumentation with geomedia is also reflected in the survey among their students. 95% of the 150 respondents partly or fully agreed with the statement: “The use of argumentation in teaching and studying promotes critical thinking and the formation of opinions”. With regard to the statement “Argumentation contributes to a mature use of digital geomedia”, 82% agreed. Consequently, students to a large extent consider argumentation as a useful method to acquire a competent use of geomedia. The statements used as a control that the use of digital media has no advantages in studies or school lessons also show that the students are convinced of the great importance of digital media (Figure 3).

A total of 86% of the respondents also agreed with the statement “Argumentation should have a high significance in the study of geography/subject matter education”. Consequently, lecturers as well as students attach great importance to the use of digital geomedia and argumentation and strongly agree in this area. The situation was different regarding the question in the following chapter.

### 4.2. Self-Assessment of Skills in Argumentation with Geomedia

The discrepancies between lecturers and students in terms of performance and competence assessment are presented here based on the interview statements of the lecturers and the contrasting results of the student survey. On the part of the lecturers, there are high doubts with regard to their students’ argumentation skills with geomedia and at the same time the assumption that the self-assessment of the students in this area deviates strongly from the actual competences:

“Yes, I found it frightening, because all these students actually feel very competent when it comes to digital media, they also use a lot of it in their free time and so on, but when you take a closer look, you are actually shocked at how little criticism there is”. (Int_Col_1)

The aspect of a lack of critical faculties on the part of the students formulated here was also mentioned by other lecturers. This indicates a lack of argumentation skills, which would be necessary to be able to express justified criticism.

One interviewee states the observation that many students have deficits in writing argumentative texts and attributes this to changed modes of communication due to digitalisation:

“The way of communication that has spread, among other things, through the digitalisation of communication, among the generation below me, among the students. With the digital natives, to which I do not belong. So, it really corresponds to short messages, Instagram, all these media, which actually no longer demand reasons and coherent texts.” (Int_FfM_4)

In summary, the lecturers see a variety of deficits among the students here. This concerns a lack of critical faculties, especially in the context of media consumption, as was also made clear in the first anchor quote of this chapter. The lecturers also see deficits in argumentative writing, which they attribute, as in the anchor quote above, to the way the so-called digital natives communicate using social media (short statements, no explanations). The lecturers also noted that students lacked technical knowledge, especially in the use of software.

**Figure 4:**
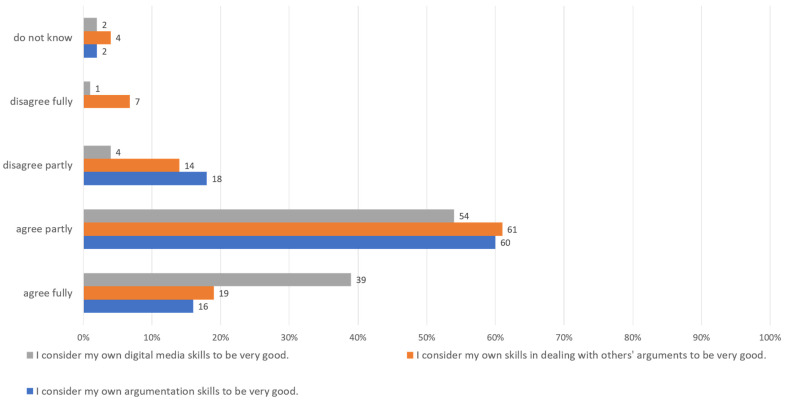
Self-assessment of students (*n* = 150) in relation to their skills in dealing with digital media, the arguments of others, and their own argumentation skills, own figure.

In contrast, the students assess themselves quite differently, both in terms of argumentation skills and in terms of their skills in dealing with digital media in general (cf. Figure 4).

However, it is obvious that these deficiencies may be related to the actual promotion of skills in courses, which is why we will take a closer look at this aspect in the following chapter.

### 4.3. Implementation and Promotion of Argumentation with Geomedia in Concrete Courses

In this section, we look at the lecturers’ statements about their own courses, and we try to make it clear, on the basis of the information about teaching that was also asked for in the interviews, which specific form of course and content is being talked about and how exactly argumentation competences are conveyed with geomedia. In contrast, we also show the results of the student survey in relation to the courses and the study programme in general. Here, discrepancies and conspicuities are again evident, especially with regard to the promotion of argumentation, because in a great number of cases this only happens implicitly, as the following two anchor quotations make clear, for example, from two GIS and cartography courses:

“And we practised that [Discussion/argumentation in the context of the course; authors’ note] a little bit, but I have to admit that we didn’t build it up systematically: how to argue conclusively, that was left to the students themselves to come up with an argumentation, we didn’t make a theoretical basis for argumentation. Maybe I would say more in the margins, then partly with maps. So how can you argue with cartography? How can I convince someone of my position with my digital geomedium, for example, through the skilful representation of layers or signatures? How is that done as well? In critical reflection.” (Int_FfM_1)

“And then you argue and discuss about it, does it belong in the map or can it be left out because it is unimportant? Is it agriculture at all? Is it part of it and similar things. So, we do talk about it, but I would now say, let’s argue, that of course not. But, yes, you compare things and ask what you think is good, what you think is bad, what you would misunderstand, you also point out gaps in the maps.” (Int_DuE_3)

It is noteworthy that in their GIS and cartography courses, the lecturers at all three university locations surveyed also pursue the goal of explaining to students the constructional character of maps and of teaching them how to argue with them or how they can be created in order to argue with them. However, as the anchor citations show, the necessary argumentation-theoretical foundations and practical skills are assumed and not explicitly addressed in the courses.

It is precisely in this area, where concrete arguments could be made with maps or digital geomedia, that linguistic support and assistance in the implementation of arguments is lacking. However, these would be necessary, as became clear in the description of deficits in the previous chapter 4.2. In fact, the lecturers interviewed seem to see the concrete promotion of argumentation competences separately from other competences and often differentiate according to seminar or course types and contents, as this statement from a lecturer who teaches GIS and cartography seminars:

“That is also in such a course, perhaps not scientific work in the sense of: how do I write a term paper, but how do I represent a certain point of view and how do I find arguments for it or against it, something like that. I believe that this also plays a role in other seminars, perhaps not so much in the more technical seminars, because the content has to be shortened a bit to accommodate more technology. So, it is not a purely technical seminar, the others are often not purely content seminars, but rather more content, here the focus is also on technology, and I have to see how much time can be allocated to one and the other? It is also difficult to combine the two.” (Int_FfM_3)

This also becomes clear in this statement concerning the seminars in physical geography, where no explicit promotion of argumentation skills is carried out, but the ability is nevertheless expected:

“So, as I said, in geomorphology there is not that much, but I also do seminars on climate history or on our future, and there we also go into a sub-political area, which is clearly better worked out, suitable for working with discussions and argumentation. We have actually already worked with the fact that written reference should be made to each other and of course the structure of individual aspects, i.e., of arguments, is not unimportant, but so clearly as a point, ok we are now working on this and we are working on argumentation, I haven’t done that yet.” (Int_Col_2)

These anchor quotations show that although argumentation is very often implicitly thought of and expected, it is not implemented in a concrete way. Only in the statements about a lecture in population and settlement geography in Cologne, where there is a focus on argumentation in research and teaching, were concrete promotions described:

*“[…] that is why my approach, especially in the first semester, is to do another introductory course on argumentation and to run it parallel to my lecture on human, population and settlement geography, and that looks like this: in every lecture I set an argumentation task on the contents of the lecture. This serves the purpose that the students repeat the contents and then also improve their argumentation skills.” (Int_Col_1)*

**Figure 5:**
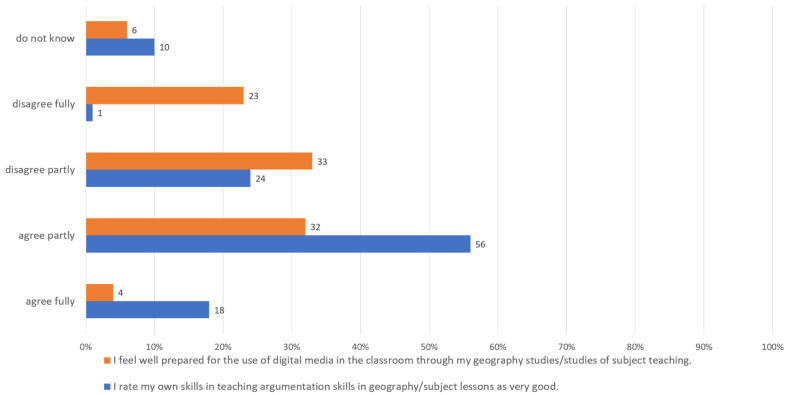
Student (*n* = 150) assessment of their training in argumentation and in the use of digital geomedia, own figure.

Here again, however, the technical component or the reference to digital geomedia is missing. In this respect, there seems to be a deficit in the training, because few concrete statements were made in the interviews that provide for the promotion of argumentation with digital geomedia. However, this would be appropriate in order to develop the student’’ skills in this area. Interestingly, the student assessment was more differentiated here (Figure 5):

The respondents rate their own abilities to teach argumentation skills in the school classroom rather well overall. This may be related to the fact that argumentation is largely only implicitly addressed and promoted. In this respect, it cannot be assumed that the respondents were actually able to give a realistic assessment here, especially against the background of the lecturer statements, who on the contrary saw deficits in this area. With regard to dealing with digital media in general, it seems that the respondents are more sceptical overall and do not necessarily see themselves as sufficiently prepared. Here, the student assessment seems to be more in line with that of the lecturers. It is also possible that students are more able to classify their own abilities in this area. The deficits in this area mentioned by the lecturers seem to be more clearly assessable here, closer to their own life reality. Starting points to promote both would have to be discussed in this respect, which will be done in the following chapter.

## 5. Discussion

The results show that lecturers and students attach great importance to digital media and argumentation with digital geomedia overall. This is consistent with the fact that the use of such media is now part of everyday life and also has high importance for science and education [8,9,28]. In this respect, it is not surprising that students and lecturers attach great importance to the subject area overall. The opinions in relation to the importance are therefore not very far apart, but in relation to the self-assessment of the students or the assessment of the students by their lecturers, this looks different. Regarding skills, the lecturer assessments are very similar to those from various studies that attest to student deficits, especially in the area of argumentation in written work [10,11] as well as in dealing with argumentation and geomedia [15]. The lecturers also perceived deficits in student writing skills, which were attributed to the way they communicate in digital media. This aspect is also in line with the findings of other studies [44] (p. 3), but could be further investigated because these are also based on the assessment of the teachers and do not yet examine the actual correlations. In contrast, the overall better self-assessment of the student’ handling of argumentation contrasts with the assessment of their lecturers. A possible explanation for this could be that the students’ inflated self-assessment also stems from the fact that their concept of argumentation follows an everyday understanding and has no clear definition. This deficit of the students is also identified elsewhere; they find it difficult to recognise assignments of meaning and to argue in a subject-related correct way instead of falling back on pre-subject-related everyday knowledge [45] (p. 55). With regard to the use of geomedia, one explanation for the overall relatively good self-assessment would be that students understand the use of geomedia to mean in particular the application area, whereas lecturers understand it to mean critical and reflective consumption. However, this aspect was not remedied here, so this would be a possible approach for a follow-up study. This is all the more significant as teacher training is a central factor for the integration of digital geomedia in the classroom [46]. So far, there is a lack of concrete, systematic promotion of argumentation competences in teacher training at the surveyed German universities for the subjects of geography and subject-specific education. The few approaches that go beyond implicit promotion (in the form of discussions) in turn do not make a symbiosis between the promotion and use of argumentation skills on the one hand and the use of digital geomedia on the other. This would be desirable, however, especially in dealing with such media, because the interviews with lecturers showed that students have major deficits here.

## 6. Conclusions

In summary, the survey shows that overall, the respondents have a positive attitude towards digital (geo)media and argumentation, and to the combined use of both, and rate the importance of both areas as high. However, on the question of student abilities, self-assessments of students diverge greatly from the assessments of their lecturers, with lecturers seeing considerable deficits. At the same time, there are hardly any concrete approaches to counteracting this and improving education. The BMBF project “Generalisability and Transferability of Digital Subject Concepts Using the Example of Digital Geomedia Use in Teacher Education (DiGeo) could represent an approach to counteract the deficits and provide lecturers with concrete tools to support their students. Within the framework of this project, digital subject concepts were promoted that contributed to the understanding of responsible digital geomedia use by teachers when dealing with argumentation. A total of 30 learning units for the promotion of argumentation, reflection and participation skills were created, 5 of which were dedicated to the direct promotion of the students’ individual skills, while the other 5 show student teachers how such skills can be taught to their future pupils. Further research in this area could be focused on conducting intervention studies to determine more precisely which approaches of the project framework and its learning units can be used to effectively promote argumentation skills in combination with the use of digital (geo)media in teacher education, and how uncertainties in one’s own skills when dealing with digital media can be eliminated. The results presented here show that this is urgently needed.

## Figures and Tables

**Table 1:** Overview of the sample.

Sample	Duisburg-Essen	Frankfurt am Main	Cologne	Wuppertal
size	30	23	88	9
female/male	21/9	15/8	60/28	8/1
average age	24			
median age	23			
average semester	5			

**Table 2:** Structure overview of the questionnaire.

	Instructions
	Request for personal data: Age, gender, place of study, intended degree, number of semesters, studied subject combination
Part I	Survey of attitudes towards digital media in general: 4 items on a four-point scale
Part II	Survey of attitudes and self-assessment towards digital geomedia in higher education: 14 items on a four-point scale
Part III	Questioning attitudes and self-assessment towards argumentation with digital geomedia: 25 items on a four-point scale
	End of survey

**Table 3:** Overview of the interview guideline (extract, own table).

	Introduction and General Questions about the Person and the Teaching Experience/Activity	
Part I Geomedia	What role do digital geomedia play in your courses? What potentials and opportunities do you see in the use of digital geomedia for your courses? What challenges do you see in the use of digital geomedia for your courses?	
Part II Argumentation	What does argumentation mean to you, can you define the term briefly? What is the importance of promoting argumentation in your courses? To what extent can argumentation be promoted with the help of digital geomedia? What could this look like in courses? What potential do you see in this area? What challenges and obstacles do you see in this area?	
	End of Survey	
Category	Definition	Anchor Example
Potentials	Statements that describe the potentials in the use of digital geomedia in the context of argumentation.	“*And with geomedia, yes, of course you also have to address examples of geomedia, if they are texts from social media or pictures, then you can work on them very well. I think it’s an excellent way to look at discussions and how you can or should or shouldn’t deal with them. I think it’s a great opportunity.*” (I_Col_No4)
Challenges	Statements that describe the challenges that lecturers face in courses when using digital geomedia in the context of argumentation.	“*Yes, I found it frightening, because all these students actually feel very competent when it comes to digital media, they also use a lot of it in their free time and so on, but when you take a closer look, you are actually shocked at how little criticism there is.*” (I_Col_No1)
Required competences	Statements that refer to concrete skills and competences that students need to learn in the context of argumentation with digital geomedia from the lecturer’s point of view.	“*Yes, that is an important point […] ok, where are the actual limits of this medium or how can I also participate in an argumentation by using different digital media. So that is a very important competence that the students have to learn.*” (I_Col_No1)
Existing competences	Statements that refer to concrete skills and competences that students already possess in the context of argumentation with digital geomedia from the lecturer’s point of view.	“*Intuitively, and the students themselves, as I said, have their geomedia in their pockets. And can handle it. And that is actually enough to learn or apply these critically reflective, reflexive competences. I don’t have to complete a GIS training course for that now.*” (I_FfM_No1)
Implementation/use in teaching courses	Statements in which the concrete implementation of argumentation with digital geomedia in own courses is described by the lecturers.	*“My introduction to geographical information systems always alludes to the fact that if you can create maps yourself, visualise data and use it, you can then make your own point of view much clearer,* i.e., *you can fulfil this level of argumentation much better.”* (I_Col_No1)

## Data Availability

Not applicable.

## References

[1] Engelen E., Budke A. (2020). Students’ Approaches when Researching Complex Geographical Conflicts using the Internet. J. Inf. Lit..

[2] Budke A. (2012). Diercke—Argumentation und Kommunikation, [Argumentation and Communication].

[3] Budke A., Uhlenwinkel A., Meyer C., Henry R., Stöber G. (2011). Argumentieren im Geographieunterricht—Theoretische Grundlagen und unterrichtspraktische Umsetzungen, [Arguing in geography lessons—Theoretical foundations and practical implementation]. Geographische Bildung: Kompetenzen in der Didaktischen Forschung und Praxis.

[4] Budke A. (2012). Argumentationen im Geographieunterricht, [Argumentations in Geography lessons]. Geogr. Its Didact..

[5] Budke A., Müller B., Gryl I., Kanwischer D., Schlottmann A. (2015). Nutzungskonflikte am Rhein als komplexe Mensch-Umwelt-Systeme mit Hilfe von Argumentationen erschließen, [Understanding Spatial conflicts on the Rhine as complex human-environment systems with the help of arguments]. Mensch:Umwelt:System.

[6] Budke A., Schiefele U., Uhlenwinkel A. (2010). Entwicklung eines Argumentationskompetenzmodells für den Geographieunterricht, [Development of an argumentation competency model for geography lessons]. Geogr. Ihre Didakt..

[7] Budke A., Seidel S., Budke A., Schäbitz F. (2021). Bedeutung der Argumentation im Lehramtsstudium der Geographie und des Sachunterrichts aus der Sicht von Hochschullehrenden, [Importance of argumentation in teacher training courses in geography and general studies from the point of view of university lecturers]. Argumentieren und Vergleichen: Beiträge aus der Perspektive Verschiedener Fachdidaktiken.

[8] Erduran S., Jimeneiz-Aleixandre M.P. (2007). Argumentation in Science Education.

[9] Sandoval W.A., Millwood K.A. (2005). The Quality of Student’ Use of Evidence in Written Scientific Explanations. Cogn. Instr..

[10] Lytzerinou E., Iordanou K. (2020). Teachers’ ability to construct arguments, but not their perceived self-efficacy of teaching, predicts their ability to evaluate arguments. Int. J. Sci. Educ..

[11] Abi-El-Mona I., Abd-El-Khalick F. (2011). Perceptions of the Nature and ‘Goodness’ of Argument among College Students, Science Teachers, and Scientists. Int. J. Sci. Educ..

[12] Nagel K., Reiss K. (2016). Zwischen Schule und Universität: Argumentation in der Mathematik, [Between school and university: Argumentation in mathematics]. Z. Erzieh..

[13] Gronostay D. (2019). Enhancing the Quality of Controversial Discussions via Argumentation Training—A Quasi-Experimental Study in Civic Education Classrooms. Bild. Erzieh..

[14] Uhlenwinkel A., Kanwischer D. (2013). Lernen im Geographieunterricht: Trends und Kontroversen: Geographiedidaktik, [Learning in Geography Classes: Trends and Controversies: Geography Education].

[15] Budke A., Kuckuck M. (2016). Politische Bildung im Geographieunterricht, [Political Education in Geography Education].

[16] Chase B.J. (2011). An Analysis of the Argumentative Writing Skills of Academically Underprepared College Students. Ph.D. Thesis.

[17] Basten M., Kraft A., Wilde M. (2017). Die Bedeutung der kommunikativen Einbettung für das Bewerten und schriftliche Argumentieren im Biologieunterricht, [The importance of communicative embedding for evaluation and written argumentation in biology lessons]. Bild. Erzieh..

[18] Macagno F., Mayweg-Paus E., Kuhn D. (2014). Argumentation Theory in Education Studies: Coding and Improving Students’ Argumentative Strategies. Topoi.

[19] Gryl I. (2015). A Starting Point: Children as Spatial Citizens. GI_Forum.

[20] Gryl I., Schulze U., Kanwischer D. (2013). Geomedien im Geographieunterricht, [Geomedia in geography lessons]. Geographiedidaktik.

[21] Jekel T., Sanchez E., Gryl I., Juneau-Sion C., Lyon J. (2014). Learning and Teaching with Geomedia.

[22] Budke A., Matthes E., Heinze C. (2011). Förderung von Argumentationskompetenzen in aktuellen Geographieschulbüchern, [Promotion of argumentation skills in current geography textbooks]. Aufgaben im Schulbuch.

[23] Favier T.T., van der Schee J.A. (2014). The effects of geography lessons with geospatial technologies on the development of high school students’ relational thinking. Comput. Educ..

[24] Xiang X., Liu Y. (2017). Understanding ‘change’ through spatial thinking using Google Earth in secondary geography: Understanding ‘change’ using Google Earth. J. Comput. Assist. Learn..

[25] Kysela-Schiemer G.G. E-Learning in der Lehrer/innenfortbildung—Akzeptanz und Wirksamkeit von E-Learning-Maßnahmen für Pflichtschullehrkräfte, [E-learning in teacher training—Acceptance and effectiveness of e-learning measures for compulsory school teachers]. Proceedings of the Forschungstag PHK 2016, Pädagogische Hochschule Kärnten.

[26] Persike M., Friedrich J.D. (2016). Lernen mit digitalen Medien aus Studierendenperspektive, [Learning with digital media from a student perspective]. Berl. Hochschulforum Digit..

[27] Fraillon J., Ainley J., Schulz W., Friedman T., Duckworth D. (2020). Preparing for Life in a Digital World. IEA International Computer and Information Literacy Study 2018 International Report.

[28] Stiftung B. (2017). Monitor Digitale Bildung. Digitales Lernen an Schulen.

[29] Gryl I. (2012). A web of challenges and opportunities. New research and praxis in geography education in view of current web technologies. Eur. J. Geogr..

[30] Metzger M.J., Flanagin A.J., Markov A., Grossman R., Bulger M. (2015). Believing the Unbelievable: Understanding Young People’s Information Literacy Beliefs and Practices in the United States. J. Child. Media.

[31] Stanford History Education Group Evaluating Information: The Cornerstone of Civic Online Reasoning. Executive Summary. https://sheg.stanford.edu/upload/V3LessonPlans/Executive%20Summary%2011.21.16.pdf.

[32] Deyrup M., Bloom B. The Truth Is Out: How Students REALLY Search. Accentuate the Positive. Proceedings of the Charleston Conference.

[33] Julien H., Barker S. (2009). How high-school students find and evaluate scientific information: A basis for information literacy skills development. Libr. Inf. Sci. Res..

[34] Leibniz Education Research Network Alliance (LERN) (2021). Education in a Digital World: Challenges and Potentials. https://www.leibniz-bildung.de/wp-content/uploads/2021/03/Position-Paper_DigiEd_AbstractEN.pdf.

[35] Gryl I., Jekel T. (2012). Re-centering geoinformation in secondary education: Toward a spatial citizenship approach. Cartographica.

[36] Schulze U., Gryl I., Kanwischer D. (2015). Spatial Citizenship education and digital geomedia: Composing competences for teacher education and training. J. Geogr. High. Educ..

[37] Lütje A., Budke A. (2021). “Es sind doch Begegnungen, wonach wir suchen”—Narration und Emotionalität im Geographieschulbuch, [“It’s encounters that we’re looking for”—Narration and emotionality in geography textbooks]. GW-Unterr.

[38] Budke A., Kuckuck M., Meyer M., Schäbitz F., Schlüter K., Weiss G. (2015). Fachlich argumentieren lernen. Didaktische Forschungen zu Argumentationen in den Unterrichtsfächern.

[39] Rinschede G. (2019). Geographiedidaktik, [Didactics of Geography].

[40] Herzig B., Ladel S., Knopf J., Weinberger A. (2018). Lehrerbildung in der digitalen Welt. Konzeptionelle und empirische Aspekte, [Teacher education in the digital world. Conceptual and empirical aspects]. Digitalisierung und Bildung.

[41] Kerres M. (2013). Mediendidaktik. Konzeption und Entwicklung Mediengestützter Lernangebote, [Media Didactics: Conception and Development of Media-Based Learning Opportunities].

[42] Döring N., Bortz J. (2016). Forschungsmethoden und Evaluation in den Sozial- und Humanwissenschaften, [Research Methods and Evaluation in the Social and Human Sciences].

[43] Flick U. (2011). Qualitative Forschung: Eine Einführung.

[44] Purcell K., Buchanan J., Friedrich L. (2013). The Impact of Digital Tools on Student Writing and How Writing is Taught in Schools. http://pewinternet.org/Reports/2013/Teachers-technology-and-writing.

[45] Uhlenwinkel A., Budke A., Kuckuck M., Meyer M., Schäbitz F., Schlüter K., Weiss G. (2015). Geographisches Wissen und geographische Argumentation, [Geographical knowledge and geographical reasoning]. Fachlich Argumentieren Lernen: Didaktische Forschungen zu Argumentationen in den Unterrichtsfächern.

[46] Bartoschek T., Carlos V., Jekel T., Car A., Strobl J., Griesebner G. (2013). What Happens When Teacher Training in Digital Geomedia is Over? Case Studies Analyzin Levels of Pedagogical Integration.

